# Factors of quality of care and their association with smartphone based PHR adoption in South Korean hospitals

**DOI:** 10.1186/s12911-021-01666-9

**Published:** 2021-10-29

**Authors:** Byung Kwan Choi, Young-Taek Park, Hyeoun-Ae Park, Chris Lane, Emmanuel C. Jo, Sunghong Kang

**Affiliations:** 1 Department of Neurosurgery, School of Medicine, Pusan National University, 2 Busandaehak-ro 63beon-gil, Geumjeong-gu, Busan, 46241 Republic of Korea; 2 HIRA Research Institute, Health Insurance Review and Assessment Service (HIRA), 60 Hyeoksin-ro, HIRA building 9th floor, Wonju-si, Gangwon-do 26465 Republic of Korea; 3 College of Nursing, Seoul National University, 103 Daehak-ro, Jongno-gu, Seoul, 03080 Republic of Korea; 4 Analytics and Intelligence, Health Workforce, New Zealand Ministry of Health, 133 Molesworth St, Thorndon, Wellington, 6011 New Zealand; 5 School of Medicine, University of Auckland, 85 Park road, Grafton, Auckland, 1023 New Zealand; 6 Department of Health Policy and Management, Inje University, 197 Inje-ro, Gimhae-si, Gyeongsangnam-do 50834 Republic of Korea

**Keywords:** Personal health records, Electronic medical records, Electronic health records, Information systems, Quality of care

## Abstract

**Background:**

Healthcare organizations have begun to adopt personal health records (PHR) systems to engage patients, but little is known about factors associated with the adoption of PHR systems at an organizational level. The objective of this study is to investigate factors associated with healthcare organizations’ adoption of PHR systems in South Korea.

**Methods:**

The units of analysis were hospitals with more than 100 beds. Study data of 313 hospitals were collected from May 1 to June 30, 2020. The PHR adoption status for each hospital was collected from PHR vendors and online searches. Adoption was then confirmed by downloading the hospital’s PHR app and the PHR app was examined to ascertain its available functions. One major outcome variable was PHR adoption status at hospital level. Data were analysed by logistic regressions using SAS 9.4 version.

**Results:**

Out of 313 hospitals, 103 (32.9%) hospitals adopted PHR systems. The nurse-patient ratio was significantly associated with PHR adoption (OR 0.758; 0.624 to 0.920, p = 0.005). The number of health information management staff was associated with PHR adoption (OR 1.622; 1.228 to 2.141, p = 0.001). The number of CTs was positively associated with PHR adoption (OR 5.346; 1.962 to 14.568, p = 0.001). Among the hospital characteristics, the number of beds was significantly related with PHR adoption in the model of standard of nursing care (OR 1.003; 1.001 to 1.005, p < 0.001), HIM staff (OR 1.004; 1.002 to 1.006, p < 0.001), and technological infrastructure (OR 1.050; 1.003 to 1.006, p < 0.001).

**Conclusions:**

One-third of study hospitals had adopted PHR systems. Standard of nursing care as well as information technology infrastructure in terms of human resources for health information management and advanced technologies were significantly associated with adoption of PHR systems. A favourable environment for adopting new technologies in general may be associated with the adoption and use of PHR systems.

## Background

Personal health record (PHR) systems are being introduced into healthcare organisations in recent years [1, 2]. Healthcare organisations have allowed their patients to view some information from their electronic medical records such as history of visits, medications, and laboratory test results through web-based programs or mobile applications [3, 4]. Some hospitals even allow their patients to enter their vital signs and symptoms to the system or make appointments for their next visits through mobile applications [5–7].

According to the Office of the National Coordinator for Health Information Technology in the United States, a PHR system is “an electronic application through which patients can maintain and manage their health information (and that of others for whom they are authorised) in a private, secure, and confidential environment.” [8] However, there has been no universally accepted definition of a PHR [9] and other terms such as *patient portal *[10], *mobile* or *m-health for patients* have been used interchangeably with PHR [11, 12].

Various factors such as improved patient-provider communication, patient empowerment and involvement, provider’s financial burden on PHR investments, and privacy and security issues are known to be associated with PHR system adoption [3, 5, 9]. In South Korea with the uniform pricing of health care services under the national health insurance program, the main incentives for patients to revisit healthcare facilities are the provision of high quality care or customer services. There are a large number of hospitals competing for patients, and at the same time, potential patients are not constrained by enrolment or insurance schemes to use particular health providers, and do not need to be referred to hospitals by primary care physicians. Potential patients are free to choose hospitals on the basis of their own experiences or their impressions of the quality of care in different hospitals [13–15]. Consequently, hospitals are marketed directly to potential patients by vigorously advertising how well patients are looked after and how advanced the hospital infrastructure is.

When patients think about visiting medical facilities, they tend to put a high priority on the quality of care [16–18]. Quality of care has a particularly strong effect on patients’ next visits in Korea because they can go to any medical facility they choose [13, 14]. Patients can even choose any tertiary hospital after getting a referral from a primary care provider. Hence medical facilities try their best to keep their patients, to maximise their profits in a competitive environment.

PHR systems have various advantages for both healthcare organisations and patients in terms of high quality of care and customer services [19, 20]. Thus, there are some possibilities that hospitals dedicating more attention to high quality of care are more likely to adopt PHR systems.

Managing health information is very important to achieve better quality of care [21]. However, the decision for a hospital to invest in a new technology depends on the current status of its human resources and technology infrastructure. It would be easy for hospitals with well-developed resources to decide on additional inputs or financial investments in information technology to improve quality of care and customer service. However, it would be more challenging for hospitals lacking such advanced infrastructure to choose to adopt the new technology because the effort and resources are too great.

Thus, hospitals that are active in improving the quality of care and customer service, and that have well-developed information technology infrastructure are expected to be early adopters of PHR systems. This implies that there may be a positive relationship between adoption of PHR systems and factors such as quality care activities, status of information technology infrastructure or advanced medical diagnostic technologies and equipment.

There have been few studies on the relationship between the adoption of PHR systems and these factors and most of studies were conducted at individual level and dealing with general topics such as various information technologies even if they were studied at organizational level [19, 22]. According to the Institute of Medicine’s report “To Err is Human”, most medical errors come from internal system failure, which can be remedied by implementing IT systems such as mandatory error reporting systems [23]. Several comprehensive review studies have found that various health information technologies such as computerized physician order entry systems and clinical decision support systems critically affect quality of care such as patient safety [23–26].

Several empirical studies indirectly suggest some possibility of positive relationship between adoption of PHR systems and quality care activities including information technology infrastructure or advanced medical diagnostic equipment. Symons et al. verified that hospitals adopting various IT applications had better quality of care with respect to gastrointestinal haemorrhage and acute myocardial infarction [27]. PHR systems in connection with quality of healthcare delivery have been recommended [28]. Healthcare delivery organizations have sought to adopt various strategies to promote patient and provider uptake of PHR systems [22]. According to a recent study, hospitals with a higher level of technological infrastructure adopted more electronic medical record systems [29]. Lack of information technology infrastructure has been mentioned as one of the impediments of PHR system adoption [30]. However, most previous studies on PHR adoption have focused on individual patients or primary care providers rather than organisations [31–33].

Little is known about PHR system adoption at organisational levels based on real world data. Hospitals in Korea run independently as medical providers. Given that PHR adoption with hospitals as units of analysis has rarely been studied internationally, findings from Korean healthcare can provide new insights and clues into how hospitals adopt IT and which hospitals are earlier adopters.

With this background, the objective of this study is to investigate the factors associated with the PHR system adoption by Korean hospitals. This study used secondary health insurance administrative data and empirically collected data from PHR vendors to focus on hospitals at the organisational level. The findings of this study will provide a basis for policy-makers to promote the introduction of various information technologies into healthcare services. However, this study does not attempt to examine the causality of adoption of PHR systems, but rather it explores the relationship between PHR adoption and factors such as quality of care activities and technological infrastructure of the hospitals.

## Methods

### Study setting

In Korea, there are three categories of hospitals based on the Medical Law and the National Health Insurance Law of Korea: tertiary hospitals, general hospitals, and hospitals (hereafter “small hospitals”). Tertiary hospitals have 20 or more medical specialties and provide training programs for these medical specialists. All of these tertiary hospitals are affiliated to medical schools. General hospitals should have seven or more medical specialties for hospitals with beds between 100 and 300, or nine or more medical specialties for hospitals with 300 or more beds. Small hospitals have beds between 30 and 100. The number of beds is closely related with independent variables of the study, namely characteristics of the hospitals. Thus, we standardized the independent variables with the number of beds and used the standardized variables in the analysis.

### Study design and data sources

The time horizon of the study is cross-sectional and the units of analysis are hospitals. There were a total of 273 general hospitals and 43 tertiary hospitals with more than 100 beds as of March 31, 2019 in Korea. We studied PHR systems in all of these hospitals from May 1 to June 30, 2020. Rather than sampling study hospitals, we included all hospitals to improve the statistical power and generalization of the study findings.

In this study “PHR” is defined as software that allows patients to access their electronic medical records in healthcare organisations through smartphones or mobile phones with the functionalities of booking, scheduling appointments, viewing or confirming prescription status, and viewing test results. PHR systems in this study are limited to mobile applications. The functions and features of the PHR systems are similar to those ones presented in previous studies [1, 34, 35].

**Fig. 1:**
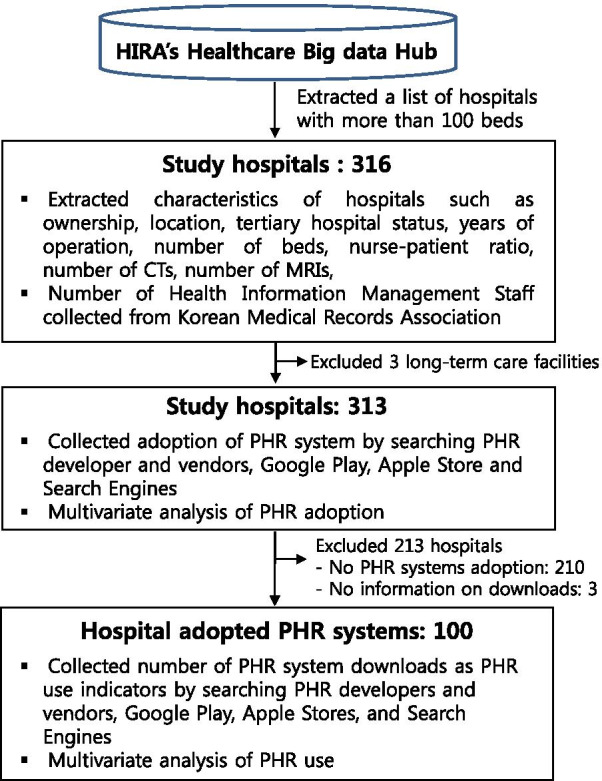
Information flows of selecting final study hospitals

Figure 1 shows a flow diagram of selecting study hospitals and collecting study variables. The list of study hospitals (including each hospital’s name and address) was extracted from the public domain website, “Healthcare Big Data Hub” (https://opendata.hira.or.kr/op/opc/selectOpenDataList.do), run by the Health Insurance Review & Assessment Services (HIRA). A researcher with a medical degree investigated the PHR adoption status of study hospitals. With the hospital lists, the researcher first conducted a market search for PHR system vendors, developers, their products, and the functionalities of the products. IT vendor companies developing PHR systems provide commercialised and customised PHR products to healthcare organizations. Thus, it was possible to obtain the list of hospitals adopting PHR systems from the vendors. For example, Lemon Healthcare, Ltd, in Korea has developed standardised mobile PHR platforms and provided them to more than 40 large hospitals.

We collected PHR adoption status from PHR vendors and online searches. First, we cross-checked the vendors’ lists of hospitals by downloading each hospital’s PHR app from Google Play and the Apple Store. Then we checked the functions of its PHR system to confirm its adoption status. Second, the researcher entered the names of the remaining hospitals (which were not in the vendor’s lists) into a search engine one by one and identified each hospital’s PHR adoption status. The search engines used were Google (google.co.kr) and a Korean search engine called NAVER (Naver.com). If it was found that a hospital had adopted a PHR system, we confirmed the adoption status by downloading its PHR app, and then recorded the functions of its PHR system.

The data collection procedure and collected functions of PHR systems are presented more in detail in a paper published by the authors [36].

The number of health information management staff (or employees) in each hospital was collected from the Korean Medical Records Association. Finally, the data were merged with the HIRA’s Healthcare Big Data Hub portal data and PHR system study data. The ethical approval for this study was obtained from the Institutional Review Board (IRB) of Pusan National University (IRB No. H-2004-026-090) on April 28, 2020. Pusan National University Hospital provided the funding for the study and was also involved in the design of the study, data collection, and revised the paper.

### Outcome and independent variables

The outcome variable is adoption of mobile-based PHR systems at hospitals. Mobile-based PHR system adoption (hereafter “PHR system adoption” or “PHR adoption”) was measured by determining whether hospitals adopted mobile PHR systems. This study included mobile PHR systems being operated on either Android or iPhone operating systems.

Three main independent variables are standard of nursing care, infrastructure capacity in terms of human resources, and advanced diagnostic technologies. Standard of nursing, a proxy variable for quality of care, was measured by the ratio of patients to nurses (nurse-patient ratio). A lower figure for the nurse-patient ratio means a higher standard of nursing care because each nurse cares for fewer patients. HIRA has introduced an incentive program for the nurse-patient ratios. There are seven grades for measuring the standard of nursing care. In the case of general hospitals, 1st grade refers to nurse-patient ratios under 2.5, 2nd grade at least 2.5 but less than 3.0, 3rd to at least 3.0 but less than 3.5, 4th to at least 3.5 but less than 4.0, 5th to at least 4.0 but less than 4.5, 6th to at least 4.5 but less than 6.0, and 7th grade to ratios of 6.0 and over. HIRA applies different reimbursement schedules to hospitals depending on these grades. This study used the same seven grades of standard of nursing care as used by HIRA. Hospital infrastructure capacity refers to resources such as buildings, human resources, equipment, IT, and processes [37]. We measured infrastructure capacity in terms of human resources and advanced diagnostic medical equipment. The former is measured by the number of health information management (HIM) staff holding a medical recorder licence and the latter is measured by the number of computerised tomographies (CTs) and magnetic resonance imaging scans (MRIs).

The remaining independent variables were extracted from publicly available open data sets in the “Healthcare Big Data Hub” run by the HIRA. The list of independent variables was affected by availability and collectability of the data. These variables are types of ownership (private versus public), location of facility (mega-metropolitan or not), types of hospitals (tertiary or general hospitals), number of years of operation, and size of hospital measured by the number of beds, which were examined in similar studies regarding PHR and adoption of electronic health record systems in the past [29, 36, 38, 39]. They were included to control the effect of confounding variables. Mega-metropolitan location is an administrative district with more than one million residents. All these data were measured as of March 31, 2020.

### Statistical analysis

This study first analysed the general characteristics of the study hospitals by PHR adoption status (no PHR adoption versus PHR adoption) using descriptive statistics. For comparing any two groups, t-tests were used for numeric measures and Chi-square tests for categorical measures.

Correlations among the independent variables were examined before a multivariate analysis. Spearman correlation was used for variables that do not follow a normal distribution. We found that the number of beds was significantly associated with the independent variables. Thus, we standardised the independent variables by the number of beds. The standardised variables were HIM staff per 100 beds, number of CTs per 100 beds, and number of MRIs per 100 beds. There was no statistically significant correlation among independent variables after standardisation by the number of beds. These adjusted figures were used in the multivariate analysis.

Logistic regression was used to investigate the relationship between PHR adoption and hospital characteristics, since the outcome variable of adopting or not adopting a PHR system is binary. SAS version 9.4 was used for the data analysis.

## Results

### General characteristics of the study hospitals

#### General characteristics of study hospitals regarding PHR adoption

Table 1 presents the general characteristics of the study hospitals. Among these hospitals, 32.9% adopted PHR systems. Hospitals that adopted PHR systems showed statistically significant differences on location (mega-metropolitan cities), tertiary hospitals, low nurse-patient ratio (meaning a high number of nurses available per patient), and high numbers of beds, CTs, and MRIs.

**Table 1:** General characteristics of the study hospitals regarding PHR adoption

Variables	Status of PHR adoption			p-value
	No PHR adoption (N = 210)	PHR adoption (N = 103)****	Total (N = 313)	
%	100.0	100.0	100.0	–
Ownership (%)				0.7012
Private	81.4	79.6	80.8	
Public	18.6	20.4	19.2	
Location (%)				0.0004
Mega-metropolitan cities	39.1	60.2	46.0	
The others	60.9	39.8	54.0	
Tertiary hospitals (%)				< 0.0001
Yes	4.8	32.0	13.7	
No	95.2	68.0	86.3	
Years of operation (std.*)	26.5 (12.4)	29.8 (15.5)	27.6 (13.5)	0.0603
Number of beds (std.)	326.8 (187.2)	641.9 (427.2)	430.4 (324.2)	< 0.0001
Nurse-patient ratio (std)**	4.3 (1.9)	2.5 (1.7)	3.7 (2.0)	< 0.0001
Number of HIM staff (std.)	4.9 (5.1)	14.2 (12.6)	7.9 (9.4)	< 0.0001
Number of CTs (std)	2.1 (1.5)	4.7 (3.4)	3.0 (2.6)	< 0.0001
Number of MRIs (std)	1.5 (0.8)	2.7 (2.3)	1.9 (1.6)	< 0.0001

*std: Standard deviation, **Seven grades based on nurse-patient ratios (see text), ***Unit: 10 thousand, ****3 cases were only iPhone operating systems

#### Correlation of study variables

Table 2 shows the correlation matrix among the independent variables after adjusting by the number of beds. No high correlation was observed among the independent variables.

**Table 2:** Correlation** among independent variables of study hospitals (N = 313)

Variables	1	2	3	4	5	*6*	*7*	*8*	*9*
1. Ownership*	1.000	0.124	− 0.089	− 0.182	− 0.030	0.024	− 0.023	0.025	0.050
		0.0285	0.1178	0.0012	0.5997	0.6689	0.683	0.6633	0.3734
2. Location*		1.000	0.172	0.087	0.102	− 0.332	0.231	0.089	0.016
			0.0023	0.1247	0.0714	< .0001	< .0001	0.1154	0.7727
3. Tertiary* hospitals			1.000	0.232	0.580	− 0.454	0.284	0.059	− 0.233
				< .0001	< .0001	< .0001	< .0001	0.2976	< .0001
4. Years of operation				1.000	0.195	− 0.162	0.164	− 0.091	− 0.238
					0.0005	0.004	0.0036	0.1092	< .0001
5. Number of beds					1.000	− 0.603	0.360	0.016	− 0.478
						< .0001	< .0001	0.7771	< .0001
6. Nurse− patient ratio						1.000	− 0.453	− 0.233	0.122
							< .0001	< .0001	0.0308
7. HIM staff***							1.000	0.286	− 0.015
								< .0001	0.7874
8. Number of CTs***								1.000	0.336
									< .0001
9. Number of MRIs***									1.000

*Binary variables were coded as 0 versus 1 such as no 1 (Private hospitals as 1,), 2 (Mega-metro cities as 1), 3 (Tertiary hospital as 1). **Spearman correlation. ***Variables 7–9 were standardised by the number of beds (each variable/bed*100)

### .Factors associated with adoption and use of PHR

#### Standard of nursing care

Table 3 shows the relationship between adoption and use of PHR systems with standard of nursing care. Interestingly, the nurse-patient ratio was significantly associated with the adoption of PHR systems after controlling for the hospital characteristics. The odds of adopting a PHR system decreases by 24.2% as the nurse-patient ratio increases (meaning that standard of nursing care deteriorated because a nurse had to take care of more patients) for one unit (OR 0.758; 0.624 to 0.920, p = 0.005).

**Table 3:** Standard of nursing care as a factor associated with PHR adoption

Variables	Adoption (N = 313)			
	OR	95% CI		p-value
		LL	UL	
Private ownership (Ref = Public)	1.048	0.494	2.224	0.903
Mega-metropolitan city location (Ref = The others)	1.589	0.880	2.868	0.124
Tertiary hospital (Ref = The others)	0.848	0.272	2.639	0.776
Years of operation	0.950	0.975	1.016	0.663
Number of beds	1.003	1.001	1.005	< 0.001
Nurse-patient ratio	0.758	0.624	0.920	0.005

Excluded three cases with iPhone operating systems because of no information on use

*OR* Odds ratio, *CI* Confidence Interval, *LL* Lower limit, *UL* Upper limit

#### HIM staff

Table 4 presents the association between adoption of PHR systems with HIM manpower in hospitals. Interestingly, the number of staff working for health information management was significantly associated with PHR adoption after controlling for the hospital characteristics. The odds of adopting a PHR system increased by 62.2% (OR 1.622; 1.28 to 2.141, p = 0.001) as the number of staff in health information management increased by one unit after controlling for the hospital characteristics.

**Table 4:** Number of HIM staff as a factor associated with PHR adoption

Variables	Adoption (N = 313)			
	OR	95% CI		p-value
		LL	UL	
Private ownership (Ref = Public)	1.071	0.945	2.318	0.862
Mega-metropolitan city location (Ref = The others)	1.741	0.976	3.106	0.060
Tertiary hospital (Ref = The others)	0.772	0.246	2.426	0.658
Years of operation	0.995	0.974	1.016	0.631
Number of beds	1.004	1.002	1.006	< 0.001
HIM staff	1.622	1.228	2.141	0.001

Excluded three cases with iPhone operating systems because of no information on use

*OR* Odds ratio, *CI* Confidence Interval, *LL* Lower limit, *UL* Upper limit

#### Technological infrastructure: CTs and MRIs

Table 5 shows the analysis results on the relationship between adoption of PHR systems with technological infrastructure in terms of CTs and MRIs. Technological infrastructure in terms of CTs and MRIs was measured by the number of CTs and MRIs per 100 beds, respectively. The number of CTs was significantly associated with PHR system adoption after controlling for the hospital characteristics (OR 5.346; 1.962 to 14.568, p = 0.001).

**Table 5:** Technological infrastructure—CTs and MRIs as factors associated with PHR adoption

Variables	Adoption (N = 313)			
	OR	95% CI		p-value
		LL	UL	
Private ownership (Ref = Public)	1.077	0.502	2.309	0.849
Mega-metropolitan city location (Ref = The others)	2.007	1.136	3.547	0.017
Tertiary hospital (Ref = The others)	0.692	0.220	2.179	0.529
Years of operation	0.997	0.975	1.018	0.754
Number of beds	1.050	1.003	1.006	< 0.001
Number of CTs	5.346	1.962	14.568	0.001
Number of MRIs	0.387	0.074	2.037	0.263

Excluded three cases with iPhone operating systems because of no information on use

*OR* Odds ratio, *CI* Confidence Interval, *LL* Lower limit, *UL* Upper limit

## Discussion

This study found that 32.9% of all hospitals with more than 100 beds in Korea adopted PHR systems. The standard of nursing care based on nurse-patient ratios and the numbers of CTs were significantly associated with both adoption of PHR systems. Hospitals with more HIM staff were significantly more likely to adopt PHR systems. Additionally, the number of beds was significantly associated with adoption.

The PHR adoption rate in this study (32.9%) was slightly higher than that of a European Union study. According to a European study conducted in 2018, requesting appointments and prescription renewals via PHR of primary care clinics such as general practitioners was 24% and 22%, respectively [40]. According to the Health Information National Trends Survey, 92% of participants indicated that they are interested in accessing to their health records via online personal health information, but the access rate was only 28% [10]. These findings suggest that mobile PHR systems have potential for high growth and a bright future.

For the relationship between standard of nursing care and adoption of PHR systems, it was found that hospitals with a low nurse-patient ratio would be more likely to adopt PHR systems. The findings of this study are similar to those of many previous studies showing that hospitals are adopting various information technologies in order to reduce medical errors and to improve quality of care [21, 27, 28]. At the micro level, it would be difficult to see the direct causal relationship between standard of nursing care with the adoption of PHR systems. However, from a broader organization’s perspective, hospitals making efforts to improve the standard of nursing care would be more likely to exert efforts to improve the quality of care by introducing new technologies. Directors or chief executives of hospitals having a low nurse-patient ratio may have had better experiences than those of other hospitals and clinics regarding the benefits of information technologies. Those cumulative efforts of hospitals for a long period of time might result in a positive relationship between quality of care factor and adoption of PHR systems. This might account for the significant relationship between PHR adoption and quality of care efforts.

Regarding hospital infrastructure and PHR adoption, this study found that hospitals with advanced infrastructure in terms of technological and human resources were more likely to adopt PHR systems. The findings of this study are similar to the findings of previous studies that found that hospitals having full EMR systems or higher stages of EMR adoption models also have advanced technological infrastructure such as having a computerized physician order entry system, laboratory information system, picture archiving communication system, or information technology department, and more information technology staff [29, 41]. Since the billing price to a patient for CTs is very high, it is beneficial to hospitals to manage patients’ appointments or booking schedules for CTs through PHR systems that can continually provide information to patients. However, it is not clear that having more HIM staff led to greater adoption of PHR systems or adoption of PHR systems led to more hiring of HIM staff, even though there is a positive relationship between PHR adoption and the number of HIM staff.

This study has several limitations. First, the unit of analysis is not patients but hospitals. In order to understand why patients are interested in using PHR systems, it is necessary to study both PHR adoption of hospitals and use with the patients as study subjects. This study did not include any patient factors in the models. Second, this study used a cross-sectional design, and as such it could not examine the causality underlying why hospitals adopt PHR systems. Further study is needed to reach a conclusion about causality by linking some organizational theories with the research findings, using more accurate and complete data.

## Conclusion

This study found that 32.9% of general and tertiary hospitals in Korea had adopted PHR systems. Hospital factors having a high standard of nursing care measured by the nurse-patient ratio, a large number of CTs, and number of beds were significantly associated with PHR adoption. A large number of HIM staff was also related with PHR system adoption. This study was conducted as an exploratory study with an intention to delve into the factors associated with PHR adoption, with the aim that health care consumers can derive more benefits from greater adoption of PHR systems.

## Data Availability

The datasets during and/or analysed during the current study are available from the corresponding author upon reasonable request.

## Abbreviations

CI: Confidence interval
CTs: Computed tomographies
HIM: Health Information Management
HIRA: The Health Insurance Review and Assessment Service
LL: Lower limit
MRI: Magnetic resonance imaging
OR: Odds ratio
PHRs: Personal health records
RR: Relative risk
UL: Upper limit
